# Efbemalenograstim alfa, an Fc fusion protein, long-acting granulocyte-colony stimulating factor for reducing the risk of febrile neutropenia following chemotherapy: results of a phase III trial

**DOI:** 10.1007/s00520-023-08176-6

**Published:** 2023-12-16

**Authors:** John Glaspy, Igor Bondarenko, Olga Burdaeva, Jianmin Chen, Dean Rutty, Renshu Li, Shufang Wang, Qingsong Hou, Simon Li

**Affiliations:** 1 UCLA School of Medicine, UCLA Medical Plaza, 100Suite 550, Los Angeles, CA 90095-6956 USA; 2 Oncology and Medical Radiology Dept, Dnepropetrovsk Medical Academy, Dnepropetrovsk, Ukraine; 3 Arkhangelsk Regional Clinical Hospital, Arkhangelsk, Russia; 4 Evive Biotechnology (Shanghai) Ltd, Shanghai, China; 5 Everest Clinical Research, Markham, ON Canada

**Keywords:** Efbemalenograstim alfa, Febrile neutropenia, Granulocyte-colony stimulating factor, Breast cancer, Docetaxel /doxorubicin therapy, Fusion protein

## Abstract

**Purpose:**

Evaluate the safety and efficacy of efbemalenograstim alfa for reducing the risk of febrile neutropenia in breast cancer patients undergoing myelosuppressive chemotherapy.

**Methods:**

A phase III, randomized, double-blind, placebo-controlled study was conducted. A total of 122 subjects received up to 4 cycles of TA chemotherapy (75 mg/m^2^ docetaxel + 60 mg/m^2^ doxorubicin). Patients were randomized in a 2:1 ratio to subcutaneously inject a single 20 mg of efbemalenograstim alfa or placebo on day 2 of cycle 1, and all subjects received efbemalenograstim alfa on day 2 of cycles 2, 3, and 4. Duration of severe (grade 4) neutropenia (DSN), depth of neutrophil nadir, incidence of febrile neutropenia (FN), time to neutrophil recovery, and safety information were recorded.

**Results:**

For the primary endpoint, the mean DSN in cycle 1 was 1.3 days and 3.9 days for efbemalenograstim alfa and placebo respectively (95% CI, 2.3, 3.4). As the lower bound of the 95% CI was > 0, superiority of efbemalenograstim alfa over placebo can be declared. In addition, the incidence of FN in Cycle 1 was lower in efbemalenograstim alfa group than in placebo group (4.8% vs. 25.6%; *p* = 0.0016). Patients in the efbemalenograstim alfa group required less intravenous antibiotics (3.6% vs. 17.9%; *p* = 0.0119). Most adverse events were consistent with those expected for breast cancer patient receiving TA chemotherapy.

**Conclusion:**

Efbemalenograstim alfa is effective and safe for significantly decreasing the duration of severe neutropenia and the incidence of febrile neutropenia in breast cancer patients who are receiving TA chemotherapy.

Trial registration.

NCT02872103, August 19, 2016.

## Introduction

Chemotherapy‐induced neutropenia can cause complications such as febrile neutropenia (FN) or other infections, which can also be life-threatening. Patients who develop FN often require prolonged hospitalizations and treatment with broad-spectrum antibiotics. The development of FN increases treatment costs and can prompt dose reductions or treatment delays, which may compromise clinical outcomes.

Guidelines recommend that risk for FN should be assessed for patients based on disease settings and chemotherapy regimens prior to treatment. Patients with high-risk for FN (> 20%) should receive prophylactic granulocyte colony-stimulating factor (G-CSF) products. Patients with intermediate-risk of FN (10–20%) should further assess the patient-specific risk factors including older age (> 65 years), prior exposure to chemotherapy or radiation therapy, persistent neutropenia, bone marrow involvement by the tumor, poor performance status, recent surgery and/or open wounds, renal or liver dysfunction, and HIV infection. If one or more of the patient-specific risk factors are met, the patient should be treated as high-risk for FN to administer G-CSFs prophylactically [1, 2].

Several G-CSF drugs have been marketed to manage neutropenia for subcutaneous administration including filgrastim [3], pegfilgrastim [4], lipegfilgrastim [5], and eflapegrastim [6] with the indication to decrease the incidence of infection, as manifested by febrile neutropenia, in patients with non-myeloid malignancies receiving myelosuppressive anti-cancer drugs associated with a clinically significant incidence of febrile neutropenia. Except for filgrastim, which is daily administered because of its 3.5 h of half-life [3], most of the G-CSFs are administered only once per chemotherapy cycle because the half-life was extended to 30–50 h through pegylated technology or fusion protein strategy. Pegfilgrastim is a PEGylated form of rh-GCSF by linking a 20-kDa PEG molecule to recombinant N-terminal methionine residue of filgrastim with half-life ranged from 15 up to 80 h. Lipegfilgrastim is a site-specific glycolpegylated r-metHu G-CSF formed by the conjugation of 20-kDa PEG-sialic acid to the O-linked glycan bound at the Thr-134 residue of G-CSF with the half-life ranged from 32 to 62 h. Eflapegrastim was developed by fusing the rhG-CSF to the Fc fragment of human IgG4 via PEG linker to increase the half-life ranged from 16.1 to 115 h [7–12].

Efbemalenograstim alfa (Ryzneuta™, F-627), is a novel Fc fusion protein, with the construct of G-CSF fused to an immunoglobulin (Ig) G2-Fc fragment via a 16 amino acid peptide linker, with decreased clearance and prolonged half-life of about 46 h. The preliminary potency of management of neutropenia has been demonstrated in the preclinical and phase II clinical studies. In vivo studies have showed that efbemalenograstim alfa may generate faster neutrophil recovery and reduce the severity of cyclophosphamide-induced neutropenia in monkeys when compared to filgrastim or pegfilgrastim [13]. In the phase II studies, efbemalenograstim alfa was shown to be statistically non-inferior to pegfilgrastim and filgrastim with respect to the mean duration of severe (grade 4) neutropenia (DSN). In the previous clinical studies, different dose levels of efbemalenograstim alfa were explored including weight-based dose of 80, 240, and 320 µg/kg and fixed dosed of 10 mg and 20 mg. Based on the study results of safety and efficacy, 20 mg fixed dose has tolerable safety and potential efficacy and was selected to further test the safety and efficacy in this study.

Here we report the results of the first pivotal phase III study of efbemalenograstim alfa (NCT02872103). This is a multicenter, randomized, double-blind, placebo-controlled study to evaluate the efficacy and safety of a single fixed dose of efbemalenograstim alfa in reducing the risk for FN in breast cancer patients receiving myelosuppressive chemotherapy.

## Methods

### Study patients

Eligible patients were females between 18 and 75 years of age, diagnosed with Stage II–IV breast cancer in the adjuvant or metastatic setting with an Eastern Cooperative Oncology Group (ECOG) performance status ≤ 2 and who were scheduled for myelotoxic TA regimen (75 mg/m^2^ Taxotere® [docetaxel] + 60 mg/m^2^ doxorubicin). Patients must have white blood cell (WBC) count ≥ 4.0 × 10^9^/L, hemoglobin ≥ 11.5 g/dL, platelet count ≥ 150 × 10^9^/L, and adequate renal, hepatic, and cardiac function. Key exclusion criteria included the following: disease progression while receiving a taxane regimen, prior treatment (within 6 weeks) with a G-CSF or a drug that may potentiate release of neutrophils, recent radiation therapy (within 4 weeks), prior chemotherapy (within 1 year), and prior bone marrow or stem-cell transplantation. Patients with a history of prior malignancy other than breast cancer may have entered the study if the malignancy was in remission and they were not receiving active treatment.

### Study design

This was a global, multi-center, randomized, double-blinded, placebo-controlled clinical study that occurred at 16 sites in the USA, Ukraine, Russia, and Hungary. Subjects were randomized 2:1 to either efbemalenograstim alfa group or placebo group using a central randomization system. Randomization was stratified by country/region to reduce regional bias. The study comprised a 14-day screening period and an 84-day treatment period.

### Procedures

Chemotherapy was administered on the first day of each 21-day chemotherapy cycle for up to 4 cycles. Approximately 24 h after chemotherapy administration in each cycle, the subject was subcutaneously injected 20 mg efbemalenograstim alfa or placebo. Subjects who randomized to placebo group received placebo injection only in cycle 1. In the following cycles 2, 3, and 4, all subjects received efbemalenograstim alfa. Efbemalenograstim alfa and placebo were provided in single pre-filled syringes.

Clinical assessments, including blood sampling, physical examination, vital signs, and symptom/toxicity assessment, occurred for all subjects during screening and at cycle-specific times throughout the study.

Oral temperature and absolute neutrophil count (ANC) behavior post-chemotherapy was tracked with daily blood draws in cycle 1 and blood draws every other day in cycles 2, 3, and 4 until ANC levels reached ≥ 2.0 × 10^9^/L, post nadir, and then 3 days later. If subjects had an ANC level < 0.5 × 10^9^/L, daily blood draws occurred until the level returned to > 0.5 × 10^9^/L. Local complete blood count were taken for safety monitoring. Peripheral blood smears to do a leukocyte differential were done and sent with the central laboratory samples.

### Efficacy assessment

The primary endpoint was the DSN in cycle 1. DSN was defined as the number of days in which the subject had an ANC < 0.5 × 10^9^/L.

Secondary endpoints included the DSN in cycles 2, 3, and 4, the time in days to ANC recovery post nadir (recovery defined as an ANC ≥ 2.0 × 10^9^/L after the expected ANC nadir) in each cycle, the depth of the ANC nadir (the lowest ANC value within 12 days chemotherapy treatment cycle) in each cycle, and the incidence of febrile neutropenia (defined as a single oral temperature of ≥ 38.3 °C [101 °F] or a.

temperature of > 38.0 °C [100.4 °F] sustained for > 1 h and ANC < 0.5 × 10^9^/L), mild to severe neutropenia, infections, and use of antibiotics in each cycle.

### Safety assessment

Safety assessments included AEs, treatment-emergent AEs (TEAEs), serious adverse events (SAEs), clinical laboratory parameters (hematology, blood chemistry, and urinalysis), vital signs, electrocardiogram parameters, and physical examinations.

Adverse events and SAEs were collected from the date of informed consent until 30 days after the completion of the study. AEs were classified by system organ class and preferred term according to the Medical Dictionary for Regulatory Activities version 20.1. The severity of AEs was graded based on the National Cancer Institute Common Terminology Criteria for Adverse Events v4.0.

All laboratory tests used for statistical analyses were performed by a designated central laboratory to ensure consistent measurements throughout the study duration.

Immunogenicity was also evaluated.

### Statistical methods

Assuming an expected difference in the DSN for efbemalenograstim alfa as compared to placebo of 2.0 days, with a common standard deviation (SD) of 3 days. Patients were randomized in a 2:1 ratio, and the dropout rate for the trial was 10%. Under these assumptions, enrollment of 80 subjects for the efbemalenograstim alfa arm and 40 subjects for the placebo group for cycle 1 would be required.

For the primary endpoint analysis, the mean DSN in cycle 1 between placebo and efbemalenograstim alfa was compared using two-sample *t*-tests at significance level of 0.05. Superiority of efbemalenograstim alfa over placebo was claimed if the lower bound of the 95% confidence interval (CI) was > 0.

Every effort was made to minimize the number of missing values for the ANC values. Local laboratory results were collected and were used for the analysis when the central laboratory ANC values were not available. Imputation methods were applied impute missing ANC value. Sensitivity analyses of pattern mixture model and tipping point analysis were used to evaluate the robustness of the primary efficacy results with respect to missing values using multiple imputation methods.

If the primary endpoint analysis inferred efbemalenograstim alfa was superior compared to placebo, then a subset of key secondary endpoints was to be tested using a fallback method to retain the type I error rate. The sequence of secondary endpoints for cycle 1 and their allocated *α* is as follows: incidence of FN (*α* = 0.04), incidence of infection (*α* = 0.005), duration of moderate neutropenia (*α* = 0.001), duration of mild neutropenia (*α* = 0.001), incidence of use of antibiotic (*α* = 0.001), incidence of use of pain medications (*α* = 0.001), and incidence of severe neutropenia (SN) (*α* = 0.001). All continuous key secondary endpoints were tested using the two-sample *t*-test, or, if the data were not normally distributed, a Wilcoxon rank-sum test. All categorical key secondary endpoints used the chi-square test to calculate *p*-values for comparisons between treatments. If the number of events in one category was < 5, Fisher’s exact test was used in place of chi-square.

The primary analysis population for all efficacy analyses was the Intent-to-Treat (ITT) analysis set, which included all randomized subjects. Safety analyses were performed on all enrolled subjects receiving any study treatment.

## Results

### Study patients

A total of 135 patients were screened and 122 subjects were randomized to the study, including 83 subjects randomized to efbemalenograstim alfa and 39 subjects randomized to placebo as their treatment in cycle 1. A single subject in the placebo group discontinued from the study during cycle 1; the subject was withdrawn from the study at the sponsor’s request as the subject was ineligible. A total of 121 subjects received 20 mg efbemalenograstim alfa during chemotherapy cycles 2, 3, and 4. Overall, 118 subjects completed the study.

Randomized subjects were predominantly White and ranged in age from 30 to 69 years. Approximately half of the subjects had stage II breast cancer, including 51.8% and 53.8% of subjects in the efbemalenograstim alfa and placebo groups, respectively. Patient demographics and baseline disease status are shown in Table 1. Similar demographic characteristics were observed in each treatment group.

**Table 1:** Patient demographics and baseline characteristics (safety population)

Characteristic	Efbemalenograstim alfa (*N* = 83)	Placebo (*N* = 39)
Age, years		
Mean (SD)	50.8 (9.25)	51.5 (9.00)
Median	50.0	51.0
Range	30, 69	33, 67
Country/region, *n* (%)		
Ukraine	46 (55.4)	22 (56.4)
Russia	32 (38.6)	16 (41.0)
Hungary	4 (4.8)	1 (2.6)
United States	1 (1.2)	0
Race, *n* (%)		
White	82 (98.8)	39 (100.0)
Black or African American	1 (1.2)	0
Baseline ECOG, *n* (%)		
0	46 (55.4)	27 (69.2)
1	37 (44.6)	12 (30.8)
BMI, kg/m^2^		
Mean (SD)	26.2 (5.36)	27.4 (6.22)
Median	26.1	26.4
Range	16, 44	17, 44
Cancer stage at screening, *n* (%)		
Stage II	43 (51.8)	21 (53.8)
Stage III	22 (26.5)	12 (30.8)
Stage IV	18 (21.7)	6 (15.4)

*BMI* body mass index, *ECOG* Eastern Cooperative Oncology Group, *SD* standard deviation, *N* number of subjects in the safety population, *n* number of subjects within a specific category

### Efficacy

#### Duration of severe neutropenia in cycle 1

As the primary endpoint, the mean (SD) DSN in Cycle 1 was 1.3 (1.19) days and 3.9 (1.44) days for efbemalenograstim alfa and placebo group, respectively (calculated with no multiple imputation; (Table 2). The mean (standard error [SE]) difference, calculated using multiple imputation for missing ANC values, was 2.9 (0.28) days (95% CI, 2.3, 3.4; *p* < 0.0001). Superiority was achieved because the lower bound of the two-sided 95% CI of the difference of placebo versus efbemalenograstim alfa > 0.

**Table 2:** Analysis of the primary efficacy endpoint, DSN in Cycle 1 (ITT Population)

	Efbemalenograstim alfa (*N* = 83)	Placebo (*N* = 39)
Mean duration (SD), days^a^	1.3 (1.19)	3.9 (1.44)
Mean difference (SE)^b^	2.9 (0.28)	
95% CI	2.3, 3.4	
*p*-value^c^	< 0.0001	

*p*-value was for the testing of mean (placebo) = mean (efbemalenograstim alfa)

*CI* confidence interval, *SD* standard deviation, *SE* standard error

^a^Mean duration of grade 4 neutropenia calculated with no multiple imputation

^b^Difference between the efbemalenograstim alfa compared to placebo (with multiple imputation)

Similar differences were observed when the analysis was performed on the ITT Population with no multiple imputation, with bootstrap sampling multiple imputation, with fully conditional specification multiple imputation, and with imputation with worst case scenario (as sensitivity analysis), and when performed on the PP population. Across these methods, the least square mean difference between efbemalenograstim alfa and placebo in the mean duration of severe neutropenia ranged between 2.6 to 2.9 days (*p* < 0.0001 when calculated). Sensitivity analyses demonstrated the robustness of the primary analysis.

##### Incidence of FN, infections, antibiotic medications and SN in cycle 1

The incidence of FN in cycle 1 was much lower in efbemalenograstim alfa group (4.8%) than in placebo group (25.6%), with a significant difference of 20.8% (*p* = 0.0016; tested at *α* = 0.04) (Table 3).

**Table 3:** Analysis of the key secondary efficacy endpoints in cycle 1(ITT Population)

	Efbemalenograstim alfa (*N* = 83)	Placebo (*N* = 39)
Incidence of FN		
n/N1 (%)	4/83 (4.8)	10/39 (25.6)
Difference with efbe (95% CI)^a^	20.8 (1.8, 38.8)	
P-value^a^(α level tested)	0.0016 (*α* = 0.04)	
Incidence of infection		
* n*/N1 (%)	2/83 (2.4)	3/39 (7.7)
Difference with efbe (95% CI)^a^	5.3 (− 13.8, 24.1)	
* p*-value^a^ (*α* level tested)	0.3258 (*α* = 0.045)	
Duration in days of grade 3 neutropenia		
* n*	74	28 5.1 (1.90)
Mean duration (SD), days	2.1 (1.51)	
Mean difference (SE)	3.0 (0.36)	
95% CI	2.2, 3.7	
* p*-value^b^ (*α* level tested)	< 0.001 (*α* = 0.001)	
Duration in days of grade 2 neutropenia		
* n*	74	28 6.8 (2.54)
Mean duration (SD), days	2.6 (1.47)	
Mean difference (SE)	4.2 (0.40)	
95% CI	3.4, 5.0	
* p*-value^b^ (α level tested)	< 0.001 (*α* = 0.002)	
Incidence of antibiotic medications		
* n*/N1 (%)	9/83 (10.8)	13/39 (33.3)
Difference with efbe (95% CI)^a^	22.5 (3.4, 40.5)	
* p*-value^a^ (*α* level tested)	0.0047 (*α* = 0.003)	
Incidence of IV antibiotic medications		
* n*/N1 (%)	3/83 (3.6)	7/39 (17.9)
Difference with efbe (95% CI)^a^	14.3 (− 4.8, 32.8)	
* p*-value^a^(*α* level tested)	0.0119 (*α* = 0.001)	
Incidence of SN		
* n*/N1 (%)	58/83 (69.9)	37/39 (94.9)
Difference with efbe (95% CI)^a^	25.0 (5.9, 42.9)	
* p*-value^a^ (*α* level tested)	0.0019 (*α* = 0.001)	

CI confidence interval, SD standard deviation, SE standard error, FN febrile neutropenia, SN severe neutropenia, IV intravenously, efbe efbemalenograstim alfa

^a^95% CI and p-value are for the proportion difference between placebo and efbemalenograstim alfa using Fisher’s exact test

^b^p-value was for the testing of mean (placebo) = mean (efbemalenograstim alfa)

^c^Post-hoc analysis

The incidence of infections in cycle 1 was 2.4% and 7.7% in efbemalenograstim alfa group and in placebo group, respectively, with a difference of 5.3% (*p* = 0.3258; tested at *α* = 0.045) (Table 3). In efbemalenograstim alfa group, 1 moderate pharyngitis and 1 mild upper respiratory tract infection were reported, s. In placebo group, 3 moderate pharyngitis and 1 moderate right pneumonia were reported. All pharyngitis resolved quickly being treated with topical antibiotics, and the pneumonia resolved being treated with 5-day oral antibiotics medication.

The incidence of antibiotic medication in cycle 1 was 10.8% and 33.3% in efbemalenograstim alfa group and in placebo group, respectively, with a difference of 22.5% (*p* = 0.0047; tested at *α* = 0.003) (Table 3).

Post-hoc analysis showed the incidence of intravenously antibiotic medication in cycle 1 was 3.6% and 17.9% in efbemalenograstim alfa group and in placebo group, respectively, with a difference of 14.3% (*p* = 0.0119; tested at *α* = 0.001) (Table 3). All intravenously antibiotic medications were used as rescue therapy when FN occurred.

The incidence of SN in cycle 1 was 69.9% and 94.9% in efbemalenograstim alfa group and placebo group respectively, with a difference of 25.0% (*p* = 0.0019; tested at *α* = 0.001) (Table 3).

The duration of grade 3 and grade 2 neutropenia was also significantly reduced in the efbemalenograstim alfa group compared to the placebo group.

##### Depth of absolute neutrophil count nadir and time to recovery in cycle 1

**Fig. 1:**
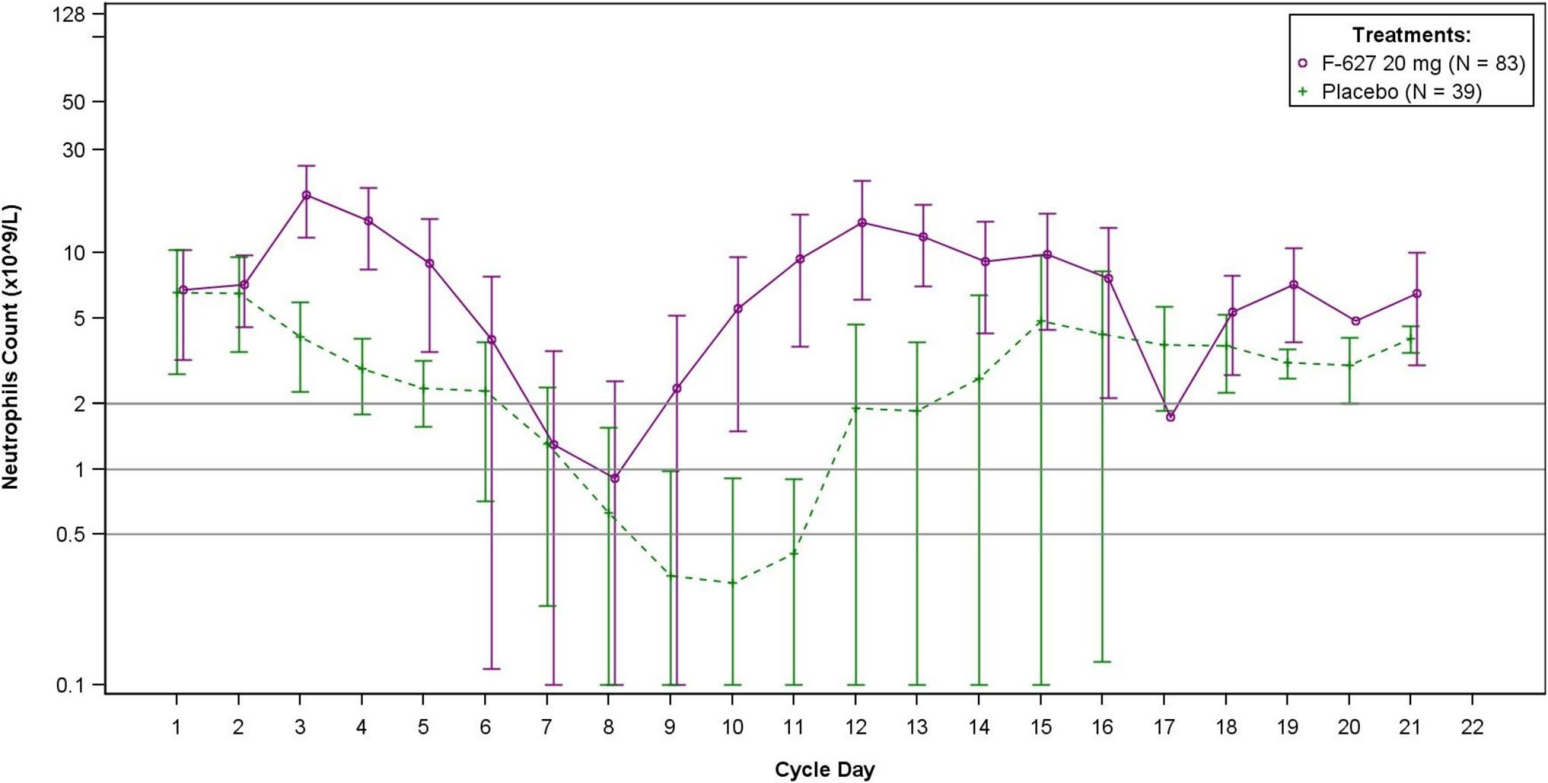
Mean absolute neutrophil counts over time in cycle 1. Abbreviations: F-627, efbemalenograstim alfa. Error bars represent standard deviation

In cycle 1, the ANC nadir was reached around day 8 and day 10 in efbemalenograstim alfa group and in placebo group, respectively (Fig. 1). The mean depth of ANC nadir was 0.7 × 10^9^/L and 0.2 × 10^9^/L, respectively, with a ratio for efbemalenograstim alfa to placebo of 3.4 (95% CI, 2.0, 5.6) (Table 4).

**Table 4:** Summary of absolute neutrophil count nadir and time to recovery post-nadir in cycle 1

	Efbemalenograstim alfa	Placebo
Depth of ANC nadir (× 10^9^/L)	N=83	N=39
Mean (SD)	0.7 (1.15)	0.2 (0.57)
Ratio to placebo (95% CI)^a^	3.4 (2.0, 5.6)	
Time to ANC recovery (days)	N=83	N=38
Mean (SD)	2.1 (1.09)	4.1 (2.06)
Mean difference (95% CI)^b^	2.0 (1.4, 2.5)	

ANC absolute neutrophil count, CI confidence interval, SD standard deviation

^a^Ratio to placebo = ANC nadir value of efbemalenograstim alfa group/ANC nadir value of placebo group. The ratio was obtained using log-transform of the data, then anti-log to get the relative effect and its 95% CI

^b^Difference between placebo to efbemalenograstim alfa

A faster ANC recovery was observed in efbemalenograstim alfa group compared to placebo group (Fig. 1). Time to ANC recovery post nadir was 2.1 days and 4.1 days, respectively, with a mean difference of 2.0 days (Table 4).

### Safety

Adverse events occurred in most of the subjects; most AEs were expected from the chemotherapy regimen such as nausea, vomiting, diarrhea, alopecia, fatigue. During cycle 1, febrile neutropenia and severe neutropenia was much more frequently observed in placebo group than in efbemalenograstim alfa group; thrombocytopenia was more frequently reported in efbemalenograstim alfa group compared to placebo group but most were mild to moderate and manageable (Table 5).

**Table 5:** Summary of adverse events in cycle 1 (incidence ≥ 5% in any treatment group) (safety population)

Adverse event	Efbemalenograstim alfa (*N* = 83)				Placebo (*N* = 39)		
	All grade	Grade 3	Grade 4	All grade		Grade 3	Grade 4
	*n* (%)	*n* (%)	*n* (%)	*n* (%)		*n* (%)	*n* (%)
Any event	74 (89.2)			36 (92.3)			
Neutropenia	54 (65.1)	11 (13.3)	39 (47.0)	25 (64.1)		0	25 (64.1)
Nausea	42 (50.6)	0	0	15 (38.5)		0	0
Leukopenia	38 (45.8)	21 (25.3)	7 (8.4)	15 (38.5)		5 (12.8)	9 (23.1)
Alopecia	33 (39.8)	4 (4.8)	0	14 (35.9)		1 (2.6)	0
Diarrhoea	17 (20.5)	1 (1.2)	0	10 (25.6)		1 (2.6)	0
Fatigue	13 (15.7)	0	0	9 (23.1)		0	0
Anaemia	12 (14.5)	1 (1.2)	0	4 (10.3)		0	0
Asthenia	10 (12.0)	1 (1.2)	0	8 (20.5)		0	0
Thrombocytopenia	10 (12.0)	1 (1.2)	0	1 (2.6)		0	0
Vomiting	10 (12.0)	0	0	8 (20.5)		0	0
Arthralgia	7 (8.4)	0	0	2 (5.1)		0	0
Stomatitis	7 (8.4)	0	0	5 (12.8)		0	0
Bone pain	6 (7.2)	0	0	4 (10.3)		0	0
Erythema	5 (6.0)	0	0	2 (5.1)		0	0
Febrile neutropenia	4 (4.8)	2 (2.4)	2 (2.4)	10 (25.6)		6 (15.4)	4 (10.3)
Decreased appetite	3 (3.6)	0	0	2 (5.1)		0	0
Headache	2 (2.4)	0	0	2 (5.1)		0	0
Dysgeusia	1 (1.2)	0	0	3 (7.7)		0	0
Pharyngitis	1 (1.2)	0	0	3 (7.7)		0	0
Gastritis	0	0	0	2 (5.1)		0	0

Bone pain, as the major consistently observed adverse event associated with G-CSF prophylaxis[11], was reported in 7.2% and 10.3% subjects in efbemalenograstim alfa group and placebo group, respectively. All bone pain were mild to moderate. In all cycles, a total of 13 subjects reported 20 events of bone pain, among them, 10 events in 6 subjects were related to efbemalenograstim alfa.

In all chemotherapy cycles, there were 17 SAEs reported in 15 (12.3%) subjects. All SAEs were assessed as unrelated to the study drug except for one event of FN that occurred on C1D9 in a subject randomized to receive placebo in cycle 1, which was assessed with a causality of unlikely. All SAEs were resolved by the end of study, with most resolving within 1–5 days of onset.

Other adverse events, which generally accepted as being attributable to G-CSF and derivatives, were not reported in this study, such as splenic rupture, sickle cell anemia with crisis, acute respiratory distress syndrome, and other events listed in the section of Precautions and Warnings on the label of G-CSFs [3–6]. Even so, these events should be carefully evaluated and monitored when using efbemalenograstim alfa.

### Immunogenicity

Among a total of 121 subjects who received at least one dose of efbemalenograstim alfa and had at least one immunogenicity data point, anti-efbemalenograstim alfa binding antibodies were detected in 11 (9.1%) subjects overall including 5 subjects positive only at baseline, 3 subjects positive at baseline and post-baseline but no treatment-boosted, and 3 subject treatment-emergent positive. No neutralizing antibodies to efbemalenograstim alfa or to G-CSF were detected.

## Discussion

Efbemalenograstim alfa is a recombinant fusion protein containing human granulocyte colony-stimulating factor and human immunoglobulin G2 (IgG2) Fc fragments, which contains 413 amino acids with a peptide linker of 16 amino acids between G-CSF and IgG2 Fc fragments. In bone marrow, it binds to G-CSF receptors stimulating progenitor cell differentiation, proliferation, and mobilization of granulocytes, including neutrophils. G-CSF receptors are present in the bone marrow regardless of the type of cancer that a patient is being treated. Similar to most marketed G-CSF products’ pivotal studies, breast cancer patients were selected as the study population in this phase III study [15–20].

As regarding to the chemotherapy in this study, TA (docetaxel/doxorubicin) chemotherapy regimen to treat breast cancer patients was selected to test the effect of efbemalenograstim alfa on neutrophils. In the guidelines [1, 2], TA, TC (docetaxel/cyclophosphamide), TAC(docetaxel/doxorubicin/cyclophosphamide) were listed as high-risk regimens for FN and selected as chemotherapy regimens in other marketed GCSF products’ pivotal studies[15–20]. In another active-controlled phase III study of efbemalenograstim alfa (NCT03252431), TC regimen to treat breast cancer patients was used to test the effect of efbemalenograstim alfa in reducing the risk of FN.

This placebo-controlled phase III study met its primary endpoint and demonstrated that efbemalenograstim alfa is superior to placebo in decreasing the duration of severe neutropenia in the first chemotherapy cycle in breast cancer patients receiving TA chemotherapy regimen. Compared to the findings of the phase III study of the first long-acting G-CSF, pegfilgrastim in breast cancer patients receiving TA chemotherapy, the mean DSN in the first cycle was similar (1.3 days versus 1.8 days) [15]. From the other efficacy endpoints analysis, efbemalenograstim alfa could reduce the incidence of FN and the incidence of infection in cycle 1 by 81% and 69%, respectively, and increase the depth of AND nadir and shorten the ANC recovery time. Primary and secondary endpoints demonstrated the potency of efbemalenograstim alfa to reduce the risk of FN and infection.

Most AEs in this study were mainly contributed to the chemotherapy. For the class effects for the G-CSF products, mild and moderate bone pains were frequently reported. In total 121 subjects who received at least one dose of efbemalenograstim alfa, only 6 (5%) subjects reported as treatment-related bone pain which was lower than commonly reported 10–35% incidence of other G-CSFs [19–21]. Due to the limited number of subjects in this study, more safety data would be obtained from the further clinical studies. Other life-threatening class of AE was not reported in this study. Safety data of this study showed that efbemalenograstim alfa was well tolerated when given subcutaneously injection at single dose of 20 mg per each chemotherapy cycle.

In conclusion, this phase III study showed that efbemalenograstim alfa is effective and safe for significantly decreasing the duration of severe neutropenia and the incidence of febrile neutropenia in breast cancer patients who are receiving TA chemotherapy. A novel, Fc fusion protein, long-acting G-CSF, efbemalenograstim alfa will expect to be an option for cancer patients with risk of chemotherapy-induced neutropenia.

## Data Availability

The data that support the findings of this study are available from the corresponding author upon reasonable request.
